# Methamphetamine Reduces LTP and Increases Baseline Synaptic Transmission in the CA1 Region of Mouse Hippocampus

**DOI:** 10.1371/journal.pone.0011382

**Published:** 2010-06-30

**Authors:** Jarod Swant, Sanika Chirwa, Gregg Stanwood, Habibeh Khoshbouei

**Affiliations:** 1 Neuroscience and Pharmacology, Meharry Medical College, Nashville, Tennessee, United States of America; 2 Department of Pharmacology, Vanderbilt University, Nashville, Tennessee, United States of America; 3 Vanderbilt Kennedy Center, Vanderbilt University, Nashville, Tennessee, United States of America; Vrije Universiteit Amsterdam, Netherlands

## Abstract

Methamphetamine (METH) is an addictive psychostimulant whose societal impact is on the rise. Emerging evidence suggests that psychostimulants alter synaptic plasticity in the brain—which may partly account for their adverse effects. While it is known that METH increases the extracellular concentration of monoamines dopamine, serotonin, and norepinephrine, it is not clear how METH alters glutamatergic transmission. Within this context, the aim of the present study was to investigate the effects of acute and systemic METH on basal synaptic transmission and long-term potentiation (LTP; an activity-induced increase in synaptic efficacy) in CA1 sub-field in the hippocampus. Both the acute *ex vivo* application of METH to hippocampal slices and systemic administration of METH decreased LTP. Interestingly, the acute *ex vivo* application of METH at a concentration of 30 or 60 µM increased baseline synaptic transmission as well as decreased LTP. Pretreatment with eticlopride (D2-like receptor antagonist) did not alter the effects of METH on synaptic transmission or LTP. In contrast, pretreatment with D1/D5 dopamine receptor antagonist SCH23390 or 5-HT1A receptor antagonist NAN-190 abrogated the effect of METH on synaptic transmission. Furthermore, METH did not increase baseline synaptic transmission in D1 dopamine receptor haploinsufficient mice. Our findings suggest that METH affects excitatory synaptic transmission via activation of dopamine and serotonin receptor systems in the hippocampus. This modulation may contribute to synaptic maladaption induced by METH addiction and/or METH-mediated cognitive dysfunction.

## Introduction

Methamphetamine (METH) is one of the most addictive drugs in existence. The illicit use of METH is a serious societal and public health problem that is rapidly accelerating [1]. Indeed, the 2006 Treatment Episode Dataset indicates the percentage of addiction treatment admissions due to METH/amphetamine abuse has risen from three percent in 1996 to nine percent in 2006 [2]. Although METH can be prescribed (to be taken PO, by mouth) to treat ADHD and obesity, drug abusers administer much larger doses [3], through faster administration routes than those used clinically. Sixty-five percent of those admitted for METH/amphetamine abuse reported smoking as the route of administration, eighteen percent reported injection, and 11 percent reported inhalation, [2]. The adverse effects of METH abuse are both short (e.g., cardiac arrhythmias, hyperthermia, insomnia, confusion [4], [5] and long-term (e.g., neurotoxicity, psychosis, cognitive impairments, addiction, changes in brain structure and function [3], [6], [7]. The neural mechanisms that underlie these behavioral responses are not completely known. Lack of such knowledge impedes evidence-based development of pharmacological intervention not only to treat addiction, but also to reverse damage caused by methamphetamine use.

METH is a substrate for the dopamine transporter and profoundly increases the concentration of extracellular monoamines dopamine (DA), serotonin (5-HT), and norepinephrine (NE) by redistributing these neurotransmitters from synaptic vesicles to the cytosol, in addition to inducing reverse transport and competing for transmitter uptake at their cognate transporters [8]. METH also affects extracellular glutamate levels [9], [10], [11]. METH is pharmacokinetically distinct from other psychostimulants- its effects are longer lasting compared to other psychostimulants such as cocaine and amphetamine, with a plasma half-life in humans of approximately 12 hours [12], [13]. Likely due to its lipophilicity, METH is widely distributed in the human and rat brain, a property it does not share with other psychostimulants such as cocaine [14], [15], [16].

The functional states of the hippocampus are under the control of many neuromodulators, including DA, 5-HT and NE. This mnemonic cortical structure receives DA input from the ventral tegmental area [17], NE input from the locus coeruleus [18], and 5-HT input from the raphe nuclei [19]. Although the hippocampus is not commonly thought to be involved in addictive behaviors, recent evidence demonstrates its involvement in psychostimulant responses and addiction. Activation of the subiculum (the main output of the hippocampal formation) has been directly implicated in the reinstatement of cocaine seeking behavior [20]. Indeed, it has recently been discovered that rats will self-administer METH directly into their hippocampus [21]. Developmental disruption of hippocampal connectivity results in increased self-administration of cocaine [22] and METH [23]. Hippocampal synaptic plasticity is bi-directionally modulated by cocaine self-administration [24]. Inactivation of hippocampal output attenuates cocaine seeking elicited by associative cues, as well as by cocaine injection [25], [26]. Because the hippocampus is implicated in the reinstatement of psychostimulant self-administration, and may even contribute to the rewarding properties of METH [21], it is critical to study the potential effects of METH in this brain region.

Drugs of abuse, including METH, can cause long-lasting changes in neuronal systems [27], [28], and alter synaptic plasticity [29], [30]. There have been a number of reports on the effects of cocaine on hippocampal synaptic transmission [24], [31], [32], [33], [34], [35], and although there are some reports concerning the effects of METH on synaptic transmission in the striatum [36] and hippocampus [37], the specific nature and pharmacology of METH-evoked changes in hippocampal plasticity are ill-defined. Here we examined the systemic and acute effects of METH on baseline synaptic transmission and long-term potentiation (LTP) in the CA1 region of the hippocampus. LTP is the potentiation of synapses caused by an induction event, such as high frequency stimulation, and is a ubiquitous model for studying long-lasting changes in the nervous system [38], [39], [40], [41], [42]. We discovered that acute *ex vivo* application of METH increased baseline excitatory synaptic transmission and decreased LTP at CA1 synapses. Furthermore, systemic administration of METH also decreased LTP. Our findings suggest that the effects of acutely applied METH on synaptic transmission appear to be mediated through the activation of both serotonergic and dopaminergic receptor systems. Some of this work has been published in abstract form [43].

## Methods

### Mice

(JAX: C57BL/6J) were housed in the AAALAC-certified animal care facility (ACF) at Meharry Medical College. Mice were maintained in an environment with ambient temperature between 22–24.5°C with a 12∶12-hour light/dark cycle and ad libitum access to food and water. For each experimental treatment mice were randomly assigned to different treatment groups. All experimental procedures complied with the NIH Guide for the Care and Use of Laboratory Animals, and were conducted with the approval of the Meharry Medical College Institutional Animal Care and Use Committee.

### Mutant mice

D1 and D5 receptor mutant mice extensively backcrossed onto C57BL/6J were obtained from Dr. Gregg Stanwood (Department of Pharmacology, Vanderbilt University Medical Center, Nashville, TN). The electrophysiological experimenter was blind to genotype. Genotype was determined before and reconfirmed by PCR after electrophysiological assessment using a previously described method [44].

### Slice preparation

**Figure 1:**
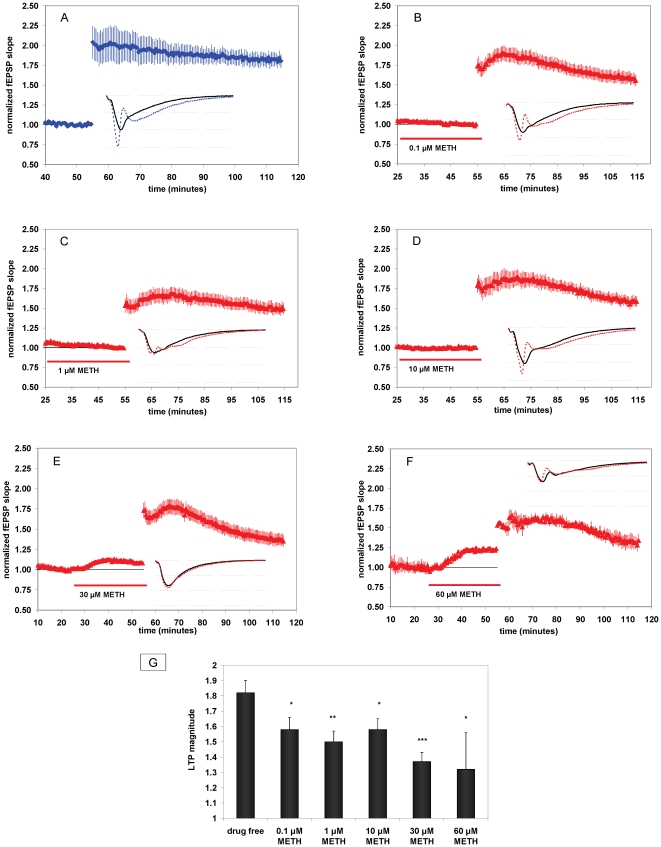
At lower concentrations, METH decreases LTP. At higher concentrations, METH increases baseline synaptic transmission and decreases LTP. (**A**) Summary plot of normalized fEPSP slope measurements recorded in the CA1 region of the hippocampus. Blue diamonds show results from drug free slices (A), red triangles (B–F) are from METH-treated slices. Error bars show ± SEM. Insets are 30-msec sweeps taken from representative experiments illustrating the average of fEPSPs 0–5 min prior to (solid line) and 55–60 min post-tetanus (dotted line, horizontal dashed lines are 1 mV apart). At 0.1 µM METH, LTP was 1.58±.08 [*n* = 13(5)] (Fig. 1B); at 1 µM METH, LTP was 1.50±.07 [*n* = 13(7)] (Fig. 1C); at 10 µM METH, LTP was 1.58±.07 [*n* = 14(8)] (Fig. 1D); at 30 µM METH, LTP was 1.37±.06 [*n* = 15(4)] (Fig. 1E); and at 60 µM METH, LTP was 1.32±.24 [*n* = 4(2)] (Fig. 1E). (**G**) **Concentration-response for the effect of METH on LTP magnitude.** * = *P*<0.05, ** = *P*<.01, *** = *P*<.001; different from drug free. Error bars depict mean ± SEM.

Acutely prepared hippocampal slices (400 µm) were obtained from anesthetized (Isoflurane) adult (2–4 months of age) mice. Transverse brain slices were dissected in ice cold, oxygenated (95% O_2_/5% CO_2_) artificial cerebrospinal fluid (aCSF) containing (mM): NaCl (125), KCL (2.5), KH_2_PO_4_ (1.25), MgSO_4_ (1.2), CaCl_2_ (2), NaHCO_3_ (25), and dextrose (10). The CA3 region was surgically removed from all slices (Fig. 1A). Slices were then transferred to a submerged recording chamber, which continuously superfused aCSF (saturated with 95% O_2_/5% CO_2_) at a rate of 1.5 ml/minute, with temperature maintained at 30°C. The slices were allowed to recover from dissection for at least an hour in the recording chamber before experiments were begun.

### Extracellular recording

Extracellular recording electrodes (borosilicate glass, ∼1 µm tip) filled with aCSF were placed in the stratum radiatum of CA1. We chose to assess field excitatory post-synaptic potentials (fEPSPs) because they provide a reliable and stable measure of excitatory synaptic transmission. Dendritic fEPSP responses were evoked with a bipolar tungsten stimulating electrode (Rhodes, Inc) placed on either the CA3 or the subicular side of the recording electrode in the stratum radiatum. Responses were amplified using the Axoclamp-2A amplifier (Molecular Devices, Inc). The electrical stimulus consisted of a single square waveform of 0.3 msec duration given at intensities of 10–130 µA generated by a Grass S88 stimulator equipped with stimulus isolation unit PSIU6.

### Data acquisition and analysis

Data were acquired with Clampex 10 and analyzed with Clampfit 10 software (Molecular Devices). The initial slope of the fEPSP (which provides a measure of the strength of excitatory synaptic transmission) was measured by fitting a straight line to the first millisecond of the fEPSP immediately following the fiber volley, and was monitored in real-time in every experiment. A stimulus-response curve was then determined using stimulation intensities between 10–130 µA. Baseline stimulation parameters were selected to evoke a response of 40–60% of the maximum slope. Baseline stimulation was then commenced at a frequency of 0.033 Hz for the entire length of the experiment. The paired-pulse protocol used in the systemic METH experiments consisted of two pulses at baseline intensity separated by 50 milliseconds. Control synaptic responses were normalized by dividing all slopes by the average of the 10 fEPSP slopes 5 minutes pre-tetanus. Since high dose METH caused a transient increase in baseline synaptic transmission, high dose METH synaptic responses were normalized by dividing all slopes by the average of the 10 fEPSP slopes 5 minutes pre-METH application. SCH23390 + METH responses were normalized by dividing all slopes by the average of the 10 fEPSP slopes 5 minutes pre-SCH23390 application. The tetanus event in the acute drug application studies consisted of four 100 Hz pulses with a 30 second inter-stimulus interval. The tetanus event in the systemic studies consisted of three 100 Hz pulses with a 30 second inter-stimulus interval. LTP was quantified as the normalized fEPSP response at 55–60 minutes post-tetanus. All baseline value comparisons were made 15–20 minutes post-drug application. Collected data was analyzed for statistical significance with an unpaired t-test, a paired t-test, or a one-way ANOVA followed by a Fisher's LSD post-hoc test, where applicable.

### Drug application

Drugs used in this project are commercially available (except for METH) from Tocris or Sigma-Aldrich. In all cases drugs were added to the aCSF for perfusion to slices. In METH only experiments, the drug was applied at t = 25 and washed out at t = 57, with tetanus at t = 55. Drug free control experiments for METH treated slices were also tetanized at t = 55. In co-application experiments, the first drug was applied at t = 25, the second at t = 55, both washed out at t = 87, with tetanus at t = 85. In control experiments for METH co-application experiments, drug was added at t = 25 and washed out at t = 87, with tetanus at t = 85. Slices taken from animals administered METH i.p. were tetanized at t = 25, and were not exposed to METH in the recording chamber.

### Systemic administration of METH

Mice were injected with 10 mg/kg METH i.p. twice daily, at 7 AM and 7 PM for five days. Mice were then sacrificed for LTP assessment approximately 16 hours after their last injection of methamphetamine. No drugs were applied to slices taken from the systemic-exposed animals.

## Results

### METH augments baseline synaptic transmission and attenuates LTP

Using *ex vivo* slice preparations derived from the CA1 region of the mouse hippocampus, we first assessed the impact of METH on synaptic plasticity. After establishing fEPSPs (average amplitude at baseline for all experiments was ∼2 mV, see sweeps in Fig. 1), we examined the effects of acute METH application on LTP. METH was administered to the slices over the range of 0.1–60 µM. The rationale for these particular concentrations derives from previous *in vivo* studies demonstrating that a 4 mg/kg i.p. injection of METH results in a concentration of about 10 µM in the mouse brain [45], whereas a 1 mg/kg i.v. infusion also results in a similar concentration in rats [46]. For humans, a dose of 30 mg i.v. is estimated to result in a concentration of 14 µM in the brain [14]. METH abusers commonly inject dosages of tens to hundreds of milligrams [3]; therefore, we investigated a wide range of METH concentrations that included low and high concentrations of METH that are within a clinically-relevant concentration range. In drug-free control slices, we observed that robust LTP was produced and maintained throughout the 60-min post tetanus. LTP magnitude 55–60 minutes post-tetanus in control slices was 1.82±.08 [*n* = 10(5)] (all *n* values are represented in the format [*n* =  number of slices (number of animals)]) (Fig. 1A). METH significantly decreased LTP compared to control at concentrations ≥0.1 µM (Fig. 1). At 30 µM and 60 µM, METH also increased baseline synaptic transmission. As shown in Fig. 1E, the magnitude of baseline synaptic responses in the apical dendritic area of the CA1, measured as changes to the fEPSP slope, increased during a 30 min application of 30 µM METH. The magnitude of normalized fEPSPs 15–20 minutes after 30 µM METH application (t = 20–25 compared to t = 40–45) was significantly increased to 1.12±.01 [*n* = 15(4)] (Fig. 1E, *P*<0.05), the fEPSP slope increased from −1.15±.10 to −1.29±.11 millivolts/millisecond (*P*<.001, paired t-test). The magnitude of normalized fEPSPs 15–20 minutes after 60 µM METH application (t = 20–25 compared to t = 40–45) was significantly increased to 1.20±.05 [n = 4(2)] (Fig. 1F, P<0.05). These changes in synaptic transmission observed at 30 µM and 60 µM METH did not occur at lower drug concentrations (Fig. 1A–D). Our data demonstrates that METH can increase glutamatergic transmission in the absence of tetanic stimulation. Since both 30 µM and 60 µM METH produced consistent and significant effects on both synaptic transmission and LTP, we chose to use the more clinically relevant concentration of 30 µM in all subsequent acute-exposure experiments.

### METH increases baseline synaptic transmission in the absence of electrical stimulation

#### METH-induced increase in synaptic transmission is transient

**Figure 2:**
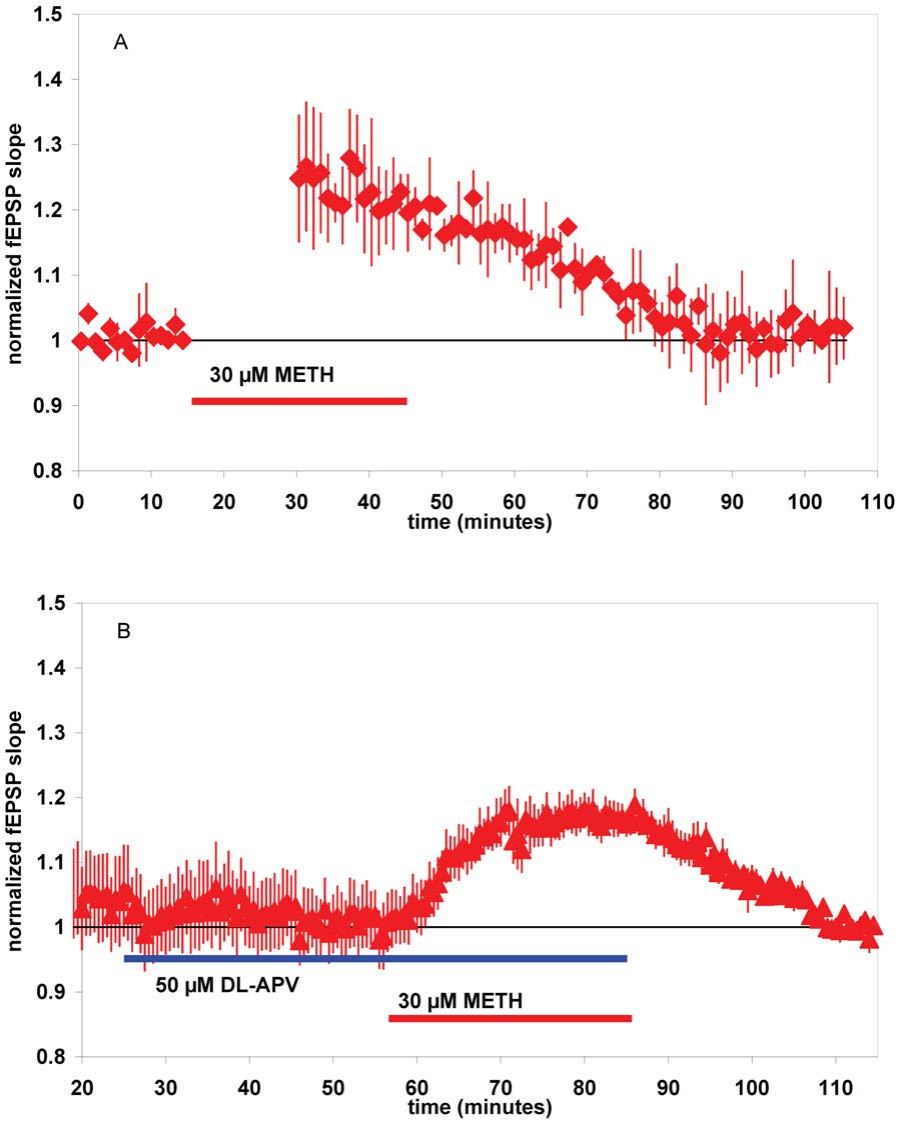
METH-induced increase in synaptic transmission does not require stimulation, and is transient- returning to baseline within and hour. Also, METH effects on baseline synaptic transmission are not dependent on NMDA receptors. (**A**) **METH increase in synaptic transmission is not long-lasting, and does not require stimulation.** Summary plot of normalized fEPSP slope measurements recorded in the CA1 region of the hippocampus. Results are from METH-treated slices. Stimulator turned off from t = 15 to t = 30. Error bars show ± SEM. (**B**) **The effects of METH on baseline synaptic transmission are not altered by blockade of NMDA receptors.** Summary plot of normalized fEPSP slope measurements recorded in the CA1 region of the hippocampus. Result are from DL-APV + METH-treated slices. Error bars show ± SEM.

Some forms of plasticity require glutamatergic activation [47]. To assess whether the observed increase of baseline synaptic transmission we observed was dependent on glutamatergic activation, we assessed the effect of 30 µM METH on fEPSPs with the stimulator turned off during the first 15 minutes of drug application. Under these conditions, METH increased fEPSPs 15–20 minutes after drug was added to the perfusion bath (Fig. 2A). Following a wash out period (as in Fig. 1, but without tetanus) we found that the effect on synaptic transmission is transient, decreasing to pre-drug baseline within ∼60 minutes (Fig. 2A). These results indicate that the correct normalization point for studies where METH induces an increase in synaptic transmission is before METH application, since the effect is gone within 60 min.

### The effects of METH on baseline synaptic transmission are not altered by blockade of NMDA receptors

LTP induction in the CA1 region of the hippocampus is predominantly mediated by NMDA receptors [48], [49], [50]. It has also been observed that METH enhances NMDA-mediated synaptic transmission [51]. To examine the possible involvement of NMDA receptors in our observed METH-induced increase in baseline synaptic transmission, we applied METH to slices pretreated with 50 µM of the NMDA receptor antagonist dl-2-amino-5-phosphonovaleric acid (DL-APV), a concentration shown previously to block NMDA-mediated currents [52]. DL-APV applied alone did not change fEPSP slopes (Fig. 2B) or affect the ability of METH (30 µM) to increase excitatory synaptic transmission. Thus, fEPSPs observed 15–20 minutes after application of DL-APV plus METH are significantly increased to 1.14±.04 [*n* = 8(4)] (Fig. 2B, *P*<0.05). These changes in fEPSP are similar to those evoked by METH alone (Fig. 1E), suggesting that the METH-evoked increase in synaptic transmission we have discovered is NMDA receptor independent.

### Effects of methamphetamine on baseline synaptic transmission and LTP are not altered by pre-application of eticlopride (a dopamine D2-like receptor antagonist)

**Figure 3:**
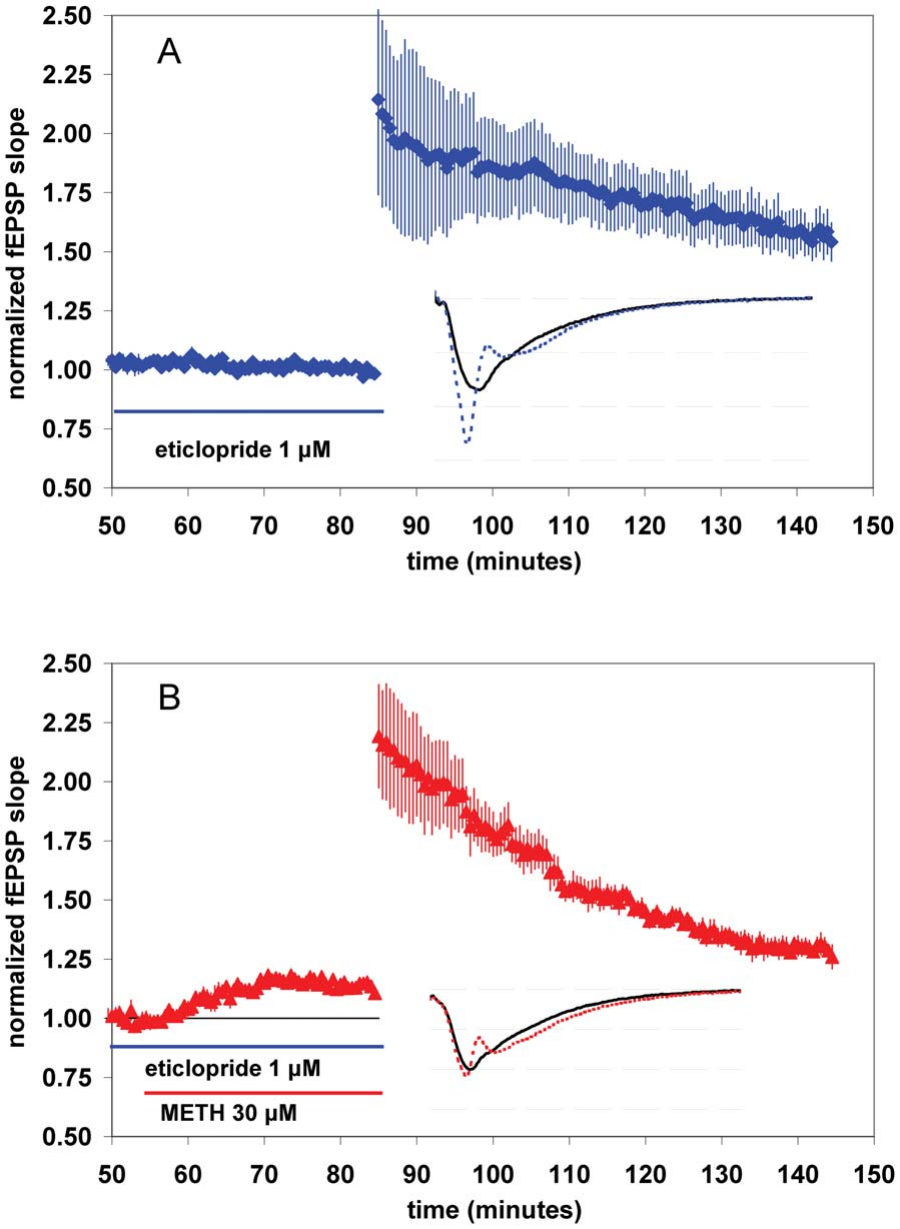
The effects of METH on baseline synaptic transmission and LTP are not altered by pre-application eticlopride (a selective D2-like dopamine receptor antagonist). (**A,B**) Summary plot of normalized fEPSP slope measurements recorded in the CA1 region of the hippocampus. The red triangles (B) are from eticlopride (1 µM) and METH (30 µM) treated slices; the blue diamonds (A) show results from eticlopride (1 µM) experiments. Error bars are ± SEM. Insets are 30-msec sweeps taken from representative experiments illustrating the average fEPSP 0–5 min prior to (solid line) and 55–60 min post-tetanus (dotted line, horizontal dashed lines are 1 mV apart).

Since METH increases the concentration of extracellular dopamine, serotonin, and norepinephrine [8], we considered the likelihood that METH's effects on baseline synaptic transmission and/or LTP could be mediated via these receptor systems. Interestingly, in the CA1 of rat hippocampus cocaine [31] and GBR12935 [53] (drugs with increase the concentration of extracellular monoamines) increase LTP by activation of D2-like dopamine receptors. Therefore, we assessed whether a D2-like receptor antagonist (eticlopride) could be effective in blocking the effects of METH. We found that eticlopride (a D2-selective receptor antagonist), at a concentration that effectively blocks a cocaine-mediated increase in hippocampal LTP [31], neither altered baseline synaptic transmission on its own, nor affected the METH-mediated increase in synaptic transmission when co-applied with METH. The normalized magnitude of fEPSPs 15–20 minutes after 1 µM eticlopride and 30 µM METH application was significantly increased to 1.16±.02 [*n* = 4(4)] (Fig. 3B, t = 50–55 compared to t = 70–75, *P*<0.05). D2-like dopamine receptor blockade also did not attenuate the effects of METH on LTP. LTP in the presence of eticlopride alone was 1.57±.09 [*n* = 3(3)], while METH still decreased LTP in slices pretreated with eticlopride (1.30±.04, [*n* = 4(4)] (Fig. 3A,B, *P*<0.05)).

### SCH23390 (a D1-like receptor antagonist) increases baseline synaptic transmission when applied alone

#### METH does not induce a further increase in synaptic transmission or a decrease in LTP after SCH23390 pretreatment

**Figure 4:**
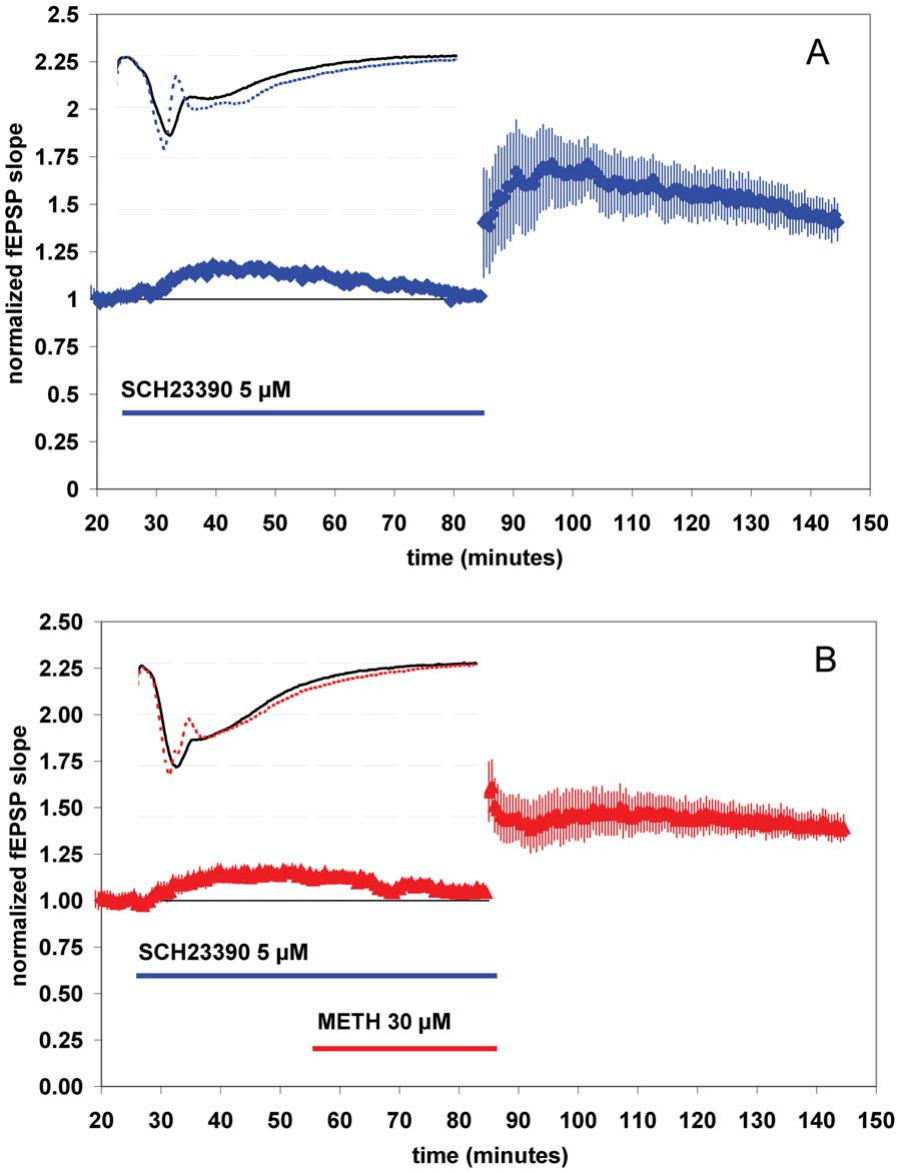
SCH23390 (a D1-like dopamine receptor antagonist) increases baseline synaptic transmission when applied alone. METH does not induce a further increase in synaptic transmission or a decrease in LTP after SCH23390 pretreatment. (**A,B**) Summary plot of normalized fEPSP slope measurements recorded in the CA1 region of the hippocampus. The red triangles (B) are from SCH23390 (5 µM) and METH (30 µM) treated slices; the blue diamonds (A) show results from SCH23390 (5 µM) experiments. Error bars are ± SEM. Insets are 30-msec sweeps taken from representative experiments illustrating the average fEPSP 0–5 min prior to (solid line) and 55–60 min post-tetanus (dotted line, horizontal dashed lines are 1 mV apart).

D1/D5 receptor agonists can induce an LTP-like potentiation in the CA1 area of the hippocampus [54], [55]. Others have demonstrated that the application of D1/D5 agonist increases LTP in the CA1 [56], [57], [58]. Since METH profoundly increases the concentration of extracellular dopamine, it is feasible that application of METH may have induced dopamine release to mediate a dopamine-receptor dependent increase in baseline synaptic transmission. To test the involvement of D1-like dopamine receptors, we utilized D1/D5-selective dopamine receptor antagonist SCH23390. Interestingly, SCH23390 increased baseline synaptic transmission when applied alone, an effect not observed with shorter application times (5–10 minutes) at the same concentration [56]. The magnitude of normalized fEPSPs 15–20 minutes after 5 µM SCH23390 application was 1.12±.03 [*n* = 7(4)] (Fig. 4A, t = 20–25 compared to t = 40–45, *P*<0.05). Furthermore, the addition of METH to the slices pretreated with SCH23390 did not induce a further increase in fEPSPs (Fig. 4B), suggesting that D1/D5 receptor activation is involved in the METH-mediated increase in baseline synaptic transmission. Consistent with previous findings [59], [60], we found that blockade of D1/D5-like receptors with SCH23390 decreased LTP. LTP was not significantly decreased by METH in slices pretreated D1/D5 antagonist compared to slices treated with SCH23390 alone. LTP in the presence of SCH23390 was 1.40±.10 [*n* = 7(4)], and 1.33±.07 [*n* = 8(4)] with SCH23390 plus METH (Fig. 4A,B).

### METH-induced increase in baseline synaptic transmission is attenuated in D1 receptor haploinsufficient mice

**Figure 5:**
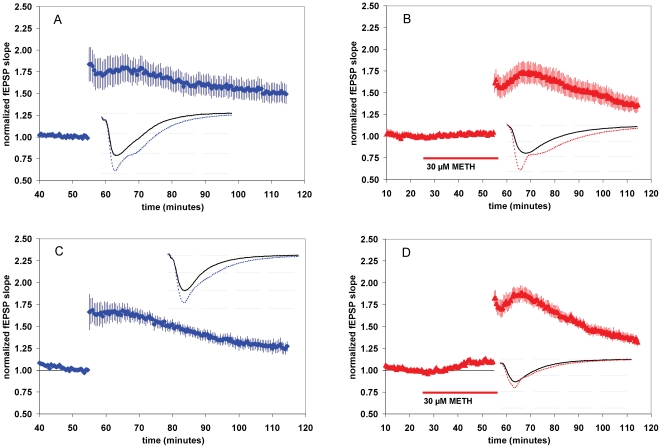
METH-induced increase in baseline synaptic transmission is attenuated in D1 receptor haploinsufficient mice. (**A,B**) Summary plot of normalized fEPSP slope measurements recorded in the CA1 region of the hippocampus of D1 +/− mice. The red triangles (B) are from METH (30 µM) treated slices; the blue diamonds (A) show results from drug-free experiments. Error bars are ± SEM. Insets are 30-msec sweeps taken from representative experiments illustrating the average fEPSP 0–5 min prior to (solid line) and 55–60 min post-tetanus (dotted line, horizontal dashed lines are 1 mV apart). (**C,D**) Summary plot of normalized fEPSP slope measurements recorded in the CA1 region of the hippocampus of D5 +/− mice. The red triangles (B) are from METH (30 µM) treated slices; the blue diamonds (A) show results from drug-free experiments. Error bars are ± SEM. Insets are 30-msec sweeps taken from representative experiments illustrating the average fEPSP 0–5 min prior to (solid line) and 55–60 min post-tetanus (dotted line, horizontal dashed lines are 1 mV apart).

**Figure 6:**
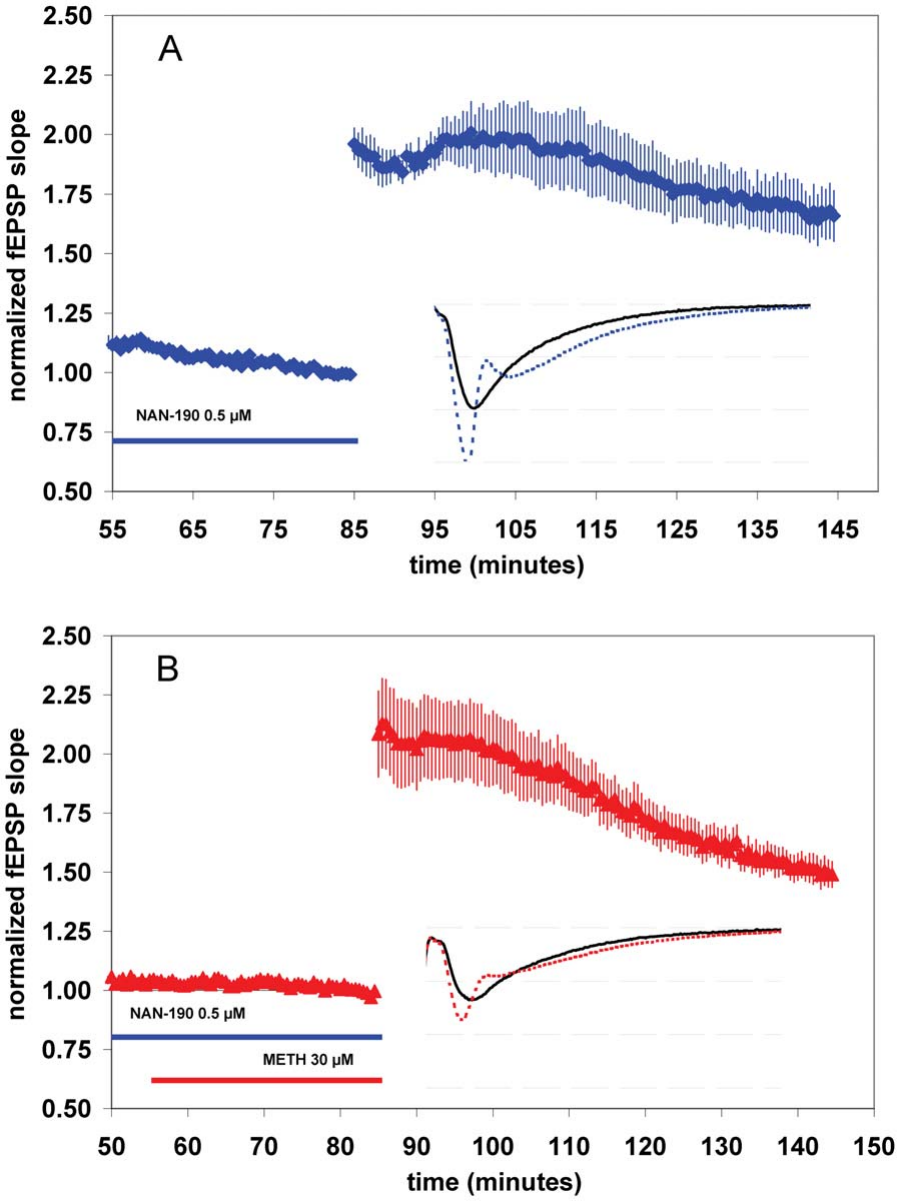
5-HT1A serotonin antagonist NAN-190 blocks the effect of METH on baseline synaptic transmission. (**A,B**) Summary plot of normalized fEPSP slope measurements recorded in the CA1 region of the hippocampus. The red triangles (B) are from NAN-190 (0.5 µM) and METH (30 µM) treated slices; the blue diamonds (A) show results from NAN-190 (0.5 µM) experiments. Error bars are ± SEM. Insets are 30-msec sweeps taken from representative experiments illustrating the average fEPSP 0–5 min prior to (solid line) and 55–60 min post-tetanus (dotted line, horizontal dashed lines are 1 mV apart).

Since no known D1 or D5-selective receptor antagonists exist, in order to further characterize the effects of METH, we utilized D1 and D5 receptor haploinsufficient mice. D1 or D5 dopamine receptor knockout/haploinsufficient mice [61], [62], [63], [64] have eliminated/reduced D1 or D5 dopamine receptor expression, respectively. We found that METH increased baseline synaptic transmission in D5 receptor haploinsufficient mice only, and was ineffective in D1 +/− mice. Normalized fEPSPs were increased 15–20 minutes after 30 µM METH in D5 +/− slices to 1.07±.01 [*n* = 6(3)] (Fig. 5D, *P*<0.05). Baseline synaptic transmission was not significantly enhanced compared to 15–20 minutes after METH in D1 receptor +/− slices to 1.03±.01 [*n* = 9(5)] (Fig. 6B, *P*<0.05). We also assessed LTP in D1 and D5 receptor haploinsufficient mice under drug free and METH-exposed conditions, finding no significant differences. LTP in drug free D5 receptor +/− mice was 1.27±.07 [*n* = 6(3)], while the addition of METH had no significant effect, resulting in an LTP magnitude of 1.23±.03 [*n* = 6(3)] (Fig. 5C,D). LTP in drug-free slices derived from D1 receptor +/− mice was 1.51±.11 [*n* = 9(5)], while the magnitude of LTP in the presence of METH decreased to 1.33±.08 [*n* = 9(5)] in D1 +/− slices (Fig. 5A,B).

### 5-HT1A serotonin antagonist NAN-190 blocks the effect of METH on baseline synaptic transmission

We examined whether the effect of METH on baseline synaptic transmission could have a serotonergic etiology, as well as a dopaminergic component. A serotonergic mechanism for the increase in baseline synaptic transmission is possible, since serotonin can reduce inhibition of excitatory synaptic transmission in the CA1 region of the hippocampus via activation of 5-HT1A receptors [65]. We observed that pretreatment with serotonin 5-HT1A competitive antagonist NAN-190 (0.5 µM) blocked the effects of METH (30 µM) on synaptic transmission. The magnitude of fEPSPs 15–20 minutes after NAN-190 and METH application was 0.99±.01 [*n* = 6(4)] (Fig. 6B, t = 50–55 compared to t = 70–75). These results suggest METH-mediated effects on glutamatergic transmission can be modulated by the serotonergic system in the hippocampus. It has been shown that the selective serotonin reuptake inhibitor fluvoxamine, which increases extrasynaptic serotonin, decreases LTP by a mechanism mediated by 5-HT1A receptors [66]. To test whether the inhibitory effect of METH on LTP also is mediated via activation of 5-HT1A receptors, we measured METH-mediated decrease in LTP when NAN-190 is co-applied. LTP in the presence of NAN-190 (0.5 µM) was 1.67±.11 [*n* = 8(6)], not significantly different from LTP in METH (30 µM) slices pretreated with NAN-190 (1.51±.06 [*n* = 6(4)] (Fig. 6A,B)). This data suggests a serotonergic component to the modulatory effect of METH in the hippocampus.

### Systemic METH exposure decreases LTP

**Figure 7:**
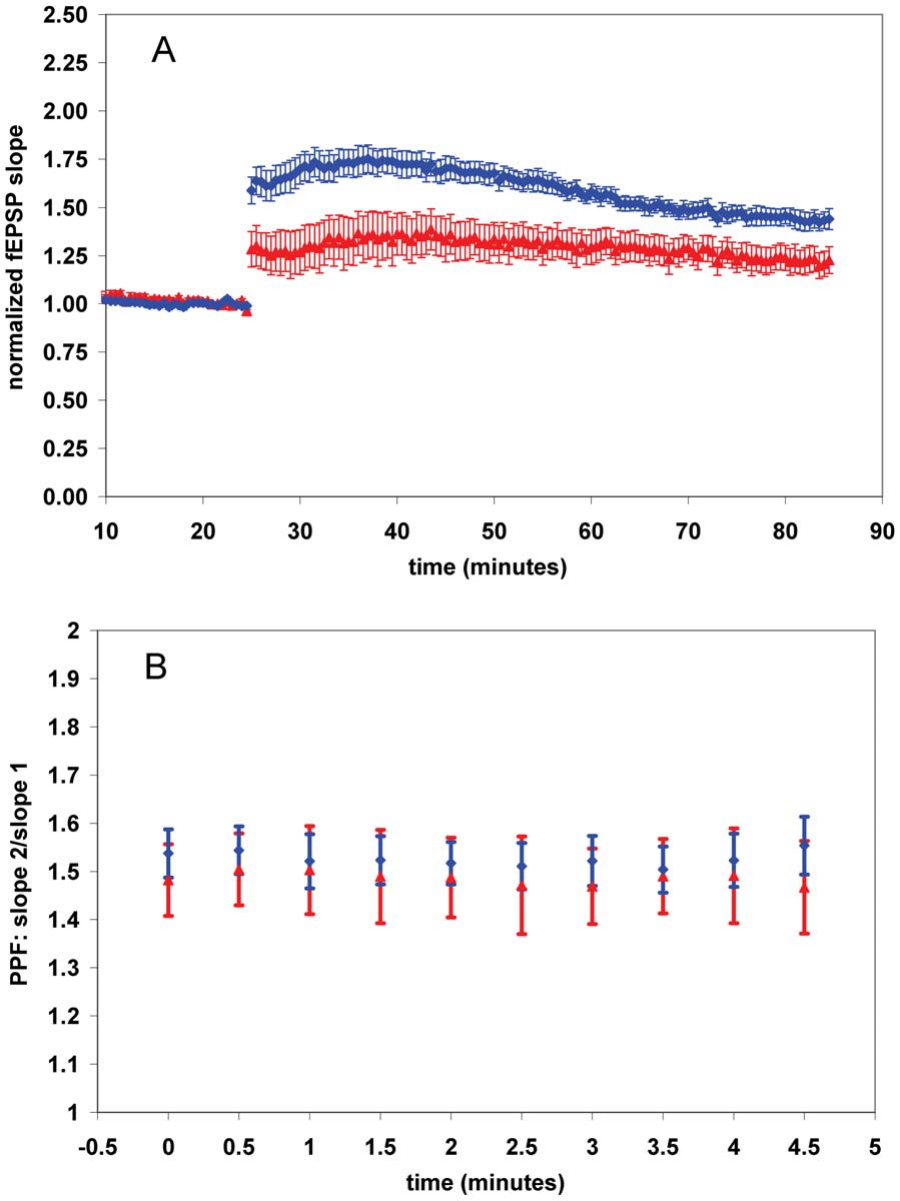
*In vivo* METH exposure also decreases LTP. (**A**) Summary plot of normalized fEPSP slope measurements recorded in the CA1 region of the hippocampus. The red triangles are from METH-treated mice; the blue diamonds show results from vehicle-treated subjects. Error bars are ± SEM. (**B**) Summary plot of paired-pulse facilitation measurements recorded in the CA1 region of the hippocampus. The red triangles are from METH-treated mice; the blue diamonds show results from vehicle-treated subjects. Error bars are ± SEM.

In addition to the acute drug exposure experiments, we tested the ability of METH to alter hippocampal synaptic plasticity in mice exposed to systemic METH. It has been shown that i.p. injection of METH decreases LTP of population spikes in CA1 [37], therefore we further assessed the effect of systemic METH exposure on hippocampal function by examining LTP of synaptic responses. Consistent with the acute-exposure studies, systemic exposure of METH resulted in deficient LTP. In vehicle-injected subjects, LTP was 1.43±.05 [*n* = 8(2)], whereas in METH-treated mice LTP (assessed 16 hours after the last METH injection) was significantly decreased to 1.22±.07 [*n* = 6(2)] (Fig. 7A). Stimulation of the CA1 Schaffer collaterals in quick succession (from tens to hundreds of milliseconds) results in the facilitation of the second synaptic response [67]. This phenomena is referred to as paired-pulse facilitation (PPF), and is thought to be due to an increase in the probability of glutamate release [68], [69] due to residual presynaptic calcium [70], [71], [72], [73]. Paired pulse facilitation was not altered by METH exposure (Fig. 7B), suggesting systemic METH does not induce a lasting change in the probability of action-potential dependent presynaptic neurotransmitter release.

## Discussion

The present study provides insight about the unique effects of METH in the hippocampus. First, acute *ex vivo* application of METH to hippocampal slices increases baseline synaptic transmission, independent of NMDA receptor activation. Second, these effects are mediated via activation of serotonergic and dopaminergic receptor systems (also see figure S1). Third, both systemic and *ex vivo* METH decrease hippocampal LTP; the systemic results in accord with a previous report from Dr. Chirwa's lab [37]. These findings contribute to our understanding of the impact of METH on hippocampal function.

We found at 30 and 60 µM METH increased baseline synaptic transmission in our preparation. This effect was not diminished by the blockade of NMDA receptors with DL-APV, indicating the observed increase in fEPSPs caused by METH is NMDA receptor-independent. Interestingly, METH also increased fEPSPs in the absence of stimulation (Fig. 2A). This may suggest the increase in synaptic transmission we have observed is induced by the action-potential independent release of monoamines [8], [74], however, action potentials occur in hippocampal slices independent of electrical stimulation [75], [76], [77]. Therefore we hypothesize that while both modes (action-potential dependent and action-potential independent) of monoamine release may occur in our hippocampal slice preparation- it is the action-potential independent mode of release that is primarily responsible for the effects of METH we have observed. Future studies will unequivocally discern whether action potentials are required for METH to increase synaptic transmission. The increase in synaptic transmission we observed was not long lasting, returning to baseline within ∼1 hour, and therefore is likely mechanistically dissimilar from LTP. Short-lived decreases in synaptic efficacy caused by dopamine, cocaine, and amphetamine have been observed in the nucleus accumbens [78]. Although qualitatively different (a decrease when we observed an increase), it is possible that the increase in synaptic transmission in the hippocampus shares a similar mechanism. Indeed, Nicola et al. [78] suggest that the decrease in synaptic transmission in the accumbens is D1-receptor mediated. Our results suggest a similar possibility.

Unlike our observed results with METH, cocaine can induce both an increase and a decrease in hippocampal LTP, depending on the concentration of acute cocaine applied [31], [35]. The decrease in LTP caused by cocaine at high concentrations may be due to blockade of sodium channels [31]. Another DAT antagonist, GBR12935, has been shown to increase LTP [53]. Thus, it was unexpected to measure a decrease in LTP mediated by METH at lower concentrations, and we tentatively hypothesized that METH may bidirectionally modulate LTP over a range of concentrations. Contrary to our hypothesis, we never demonstrated an increase in LTP in response to METH over a wide range of concentrations. Indeed, we observed a *decrease* in LTP over a range of concentrations.

Recently it has been shown that eticlopride, a D2-receptor antagonist, blocks a cocaine-induced increase in LTP, [31]. Our findings indicate that, in contrast to cocaine, the effects of METH are not mediated by the D2 dopamine receptor. As mentioned previously, it is unclear why cocaine and METH, having a similar pharmacological outcome (an increase in extracellular monoamines), have different effects on synaptic plasticity apparently mediated by different receptor systems. Indeed, the ‘cocaine paradox’ [79], in which monoamine neurotransmitter transporter blockers (e.g., cocaine, GBR12935) curiously reduce the effect of transporter substrates, or releasers (e.g., methamphetamine, amphetamine), elegantly demonstrates that METH-exposure results in a higher extrasynaptic concentration of monoamines than drugs which block monoamine neurotransmitter transporters. Why then, do we not simply see the same effects, only larger, induced by METH vs. DAT blockers? One possible explanation is that the larger sphere and duration of monoaminergic influence, or volume transmission [80] induced by METH exposure may activate different subsets of receptors. Another intriguing speculation is that cocaine and METH may have differing activity at sigma receptors, indeed, sigma receptor agonists can decrease LTP in the CA1 [81].

As previously reported, we also observed that the D1/D5 dopamine antagonist SCH23390 decreases LTP *per se* [59]. Our surprising finding that SCH23390 on its own increased baseline synaptic transmission might be explained by our longer drug treatment protocol before the tetanus stimulation (30 min) compared to previously published observations [54], [56]. The finding that application of METH after pre-application of SCH23390 did not increase in baseline synaptic transmission further may suggest that the METH-mediated modulation of fEPSPs might in part be due to the release of endogenous dopamine and subsequent activation of D1/D5-like receptors (also see figure S1). Application of D1/D5 agonists can induce a potentiation that is long lasting, D1/D5 receptor specific, and requires protein synthesis and glutamatergic activation [47], although some have not observed this phenomenon [55]. The short duration of the increase in fEPSPs induced by METH indicates it is likely not similar to the long-lasting D1/D5 agonist potentiation others have observed [54].

Since no known D1 or D5-selective pharmacological tools currently exist, D1 and D5 haploinsufficient mice are attractive tools to further characterize the putative dopaminergic effects of METH. In addition, a haploinsufficient genotype is a more generalizable condition to human pathophysiology than complete knockout of a protein [82]. The finding that METH increased baseline synaptic transmission only in D5 receptor haploinsufficient mice suggests that the effects of METH in part may rely on D1-like dopamine receptors, and in addition, specifically implicate the D1 receptor in the effects of METH on baseline synaptic transmission. Although LTP has been assessed in D1 receptor knockout mice [83], to our knowledge synaptic plasticity has not been studied in either D1 or D5 haploinsufficient mice, thus, our results provide additional information about the modulatory effects of D1 and D5 receptors on LTP. To our knowledge, we are first to show a deficit in LTP in D5 receptor +/− mice, suggesting that the D5 receptor is required for the normal expression of synaptic plasticity.

Our findings that METH-evoked effects on synaptic transmission and LTP are reduced by a 5-HT1A antagonist, NAN-190 implicate a serotonergic mechanism for METH in the hippocampus. The reported effects of 5-HT in the hippocampus are divergent, as some have documented inhibitory [84], [85], [86], while others have shown excitatory [65] effects, or no effect on synaptic transmission [87]. Our results suggest that 5-HT has an excitatory effect in the CA1 (also see figure S1). One documented mechanism for excitatory effects of 5-HT in the hippocampus suggests 5-HT1A receptor activation can inhibit tonic GABA release from inhibitory interneurons, thereby inhibiting glutamatergic transmission [65]. Future studies are required to further examine this putative METH-induced serotonergic modulation of hippocampal neurotransmission.

Our studies on the systemic exposure of animals to METH also demonstrated a decrease in LTP. We observed no change in paired-pulse facilitation, which suggests that there is not a change in the probability of transmitter release caused by systemic METH administration. It is of interest to note that the half-life of METH in mice is ∼1 hour [88], [89], [90], much shorter than in humans [12], [91], [92]. We assessed the LTP ∼16 hours after the last injection. Using a t1/2 of 70 minutes, the elimination rate constant can be easily calculated (t1/2 = ln(2)/k). Assuming instantaneous absorption, we estimated of the percent of drug plasma concentration remaining after sixteen half-lives using first order kinetics with concentration of drug at time zero being equal to 100% (C(0) = 100%) using the equation C_t_ = C_(0)_e^−kt^ (C = concentration, e = 2.7183, k = elimination rate constant, t = time). This estimate (which does not account for i.p. absorption), indicates that after 16 hours, the percentage of the initial concentration of METH remaining would be 0.0074%. We estimate that nearly all of the METH from the last i.p. injection was cleared at the time the mice were sacrificed for LTP experiments [92]. This suggests that METH has a lasting effect on synaptic plasticity after the drug is cleared from the body. It is unknown how long the effect of METH on LTP will last, or if it will remain qualitatively similar, since bi-directional modulation of synaptic plasticity by drugs of abuse (cocaine) has been observed after long periods of withdrawal [24]. This is an important question, and will be further studied.

In summary, we provide evidence that METH increases baseline synaptic transmission *(before high frequency tetanus* see Fig. 1E,F; time = 30–40 min) and decreases the magnitude of LTP induced by a high frequency tetanus event. We propose a dopaminergic and serotonergic mechanism for the effect of METH on fEPSPs. Our results also suggest that the D1 dopamine receptor may be specifically involved in the effect of METH on synaptic transmission. We hypothesize that deficient hippocampal synaptic plasticity may be a hallmark of METH-induced adverse cognitive/addictive processes.

## Supporting Information

Figure S1 Application of dopamine (DA), or serotonin (5-HT) increases baseline synaptic transmission. (A) Summary plot of normalized fEPSP slope measurements recorded in the CA1 region of the hippocampus. Green circles show results from dopamine-treated slices, purple squares are from serotonin-treated slices. Error bars show ± SEM, no tetanus was given.
(0.63 MB TIF)
