# Supplementary figures and images for: Methamphetamine Reduces LTP and Increases Baseline Synaptic Transmission in the CA1 Region of Mouse Hippocampus
Source: PLoS One. 2010 Jun 30;5(6):e11382. doi: 10.1371/journal.pone.0011382 (PMC2894864; doi:10.1371/journal.pone.0011382)

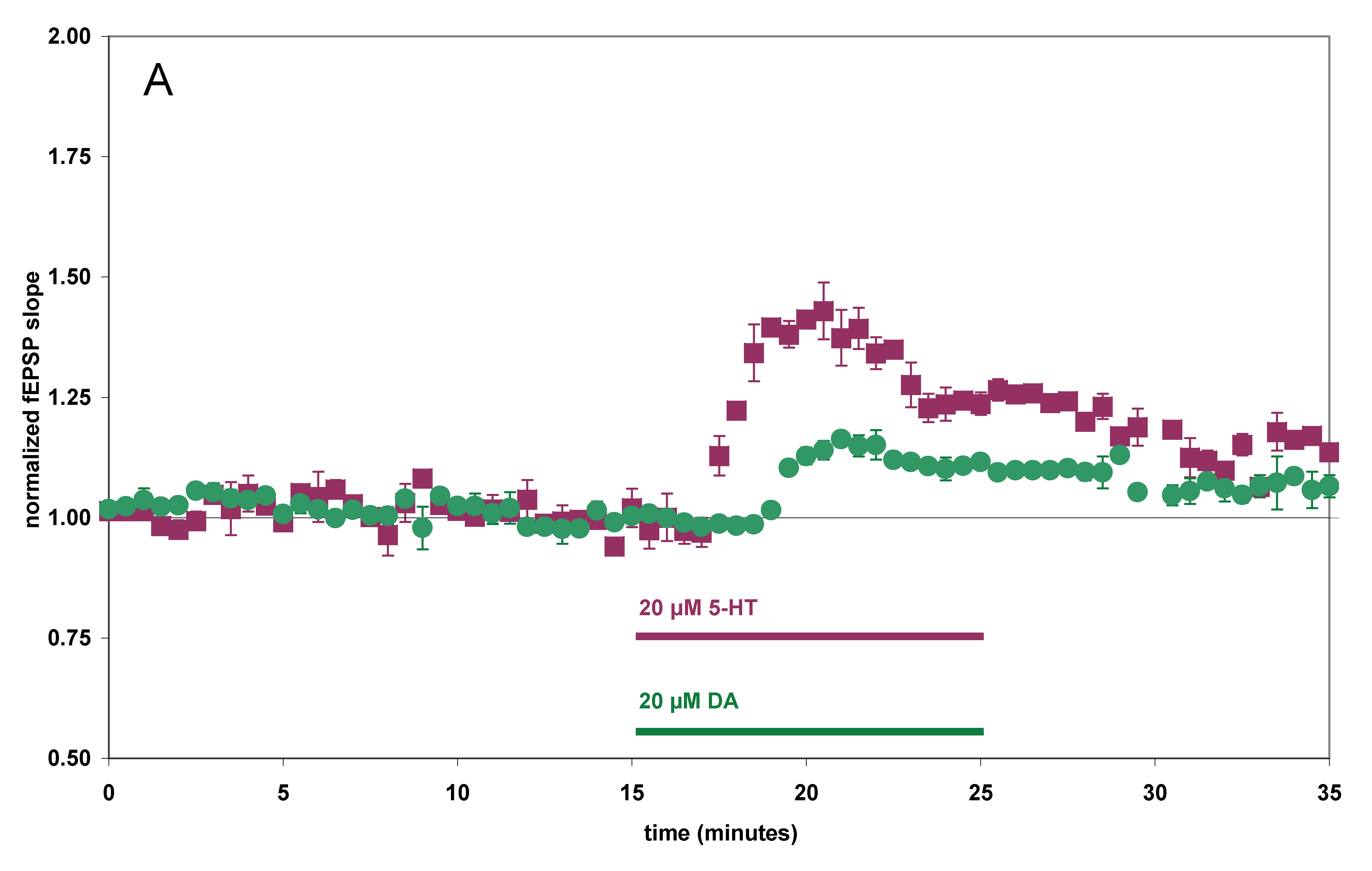

Supplement: Figure S1 — Application of dopamine (DA), or serotonin (5-HT) increases baseline synaptic transmission. (A) Summary plot of normalized fEPSP slope measurements recorded in the CA1 region of the hippocampus. Green circles show results from dopamine-treated slices, purple squares are from serotonin-treated slices. Error bars show ± SEM, no tetanus was given. (0.63 MB TIF) [file pone.0011382.s001.tif]
